# Supplementary material for: The interactome of intact mitochondria by cross-linking mass spectrometry provides evidence for coexisting respiratory supercomplexes
Source: Mol Cell Proteomics. 2017 Dec 8;17(2):216–32. doi: 10.1074/mcp.RA117.000470 (PMC5795388; doi:10.1074/mcp.RA117.000470)
Supplement: Supplemental Data [file supp_RA117.000470_133922_0_supp_23978_fzffwf.zip › spectra_annotation/mito_spectra_annotation/136-1-2-1-7-1.pdf]

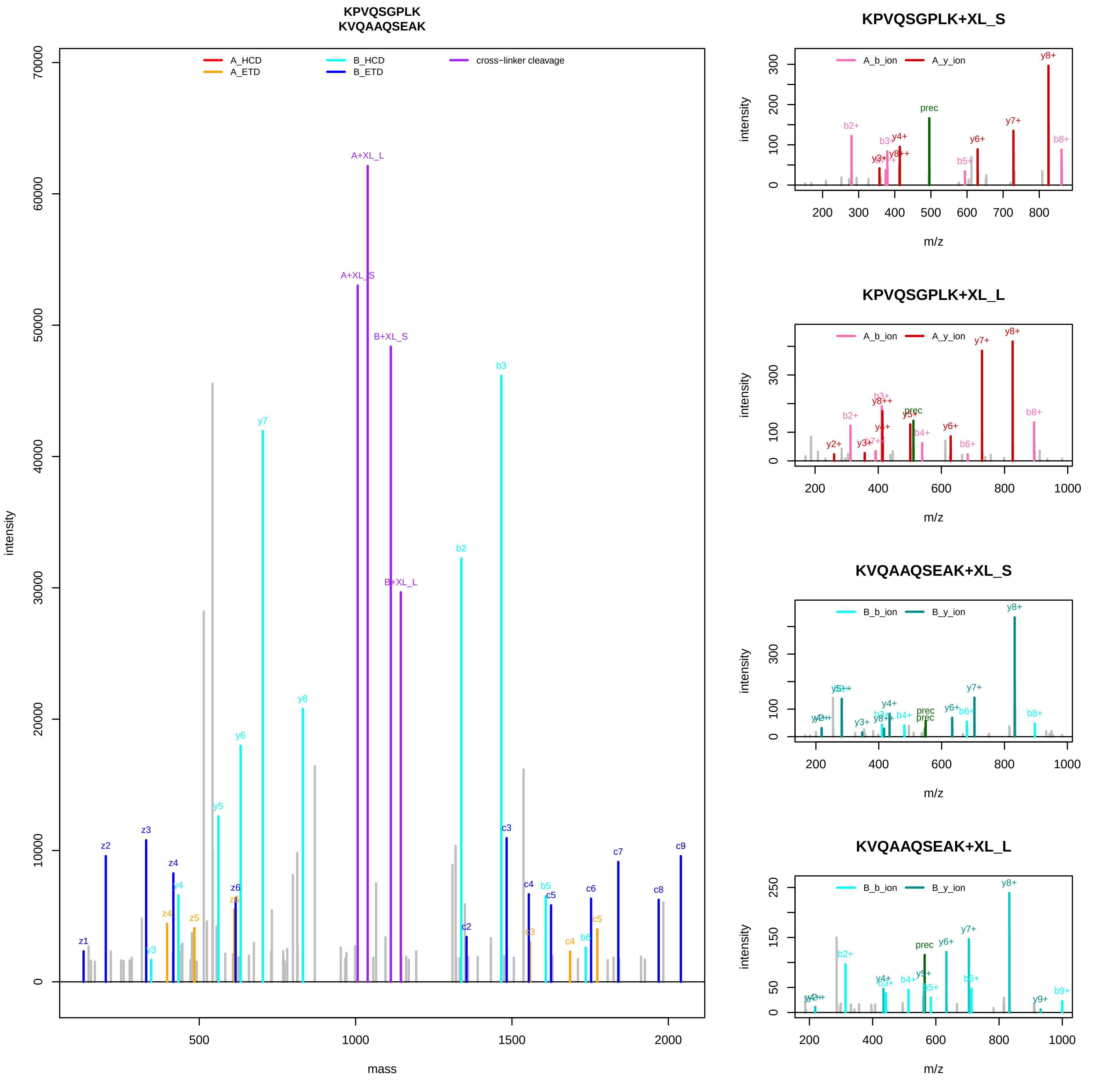

KPVQSGPLK
KVQAAQSEAK
A_HCD
A_ETD
B_HCD
B_ETD
cross−linker cleavage
A+XL_L
A+XL_S
B+XL_S
B+XL_L
b3
b2
y7
y8
y6
y5
y4
y3
z1
z2
z3
z4
z5
z6
c2
c3
c4
c5
c6
c7
c8
c9
b5
b6
intensity
mass
0
10000
20000
30000
40000
50000
60000
70000
500
1000
1500
2000
KPVQSGPLK+XL_S
A_b_ion
A_y_ion
prec
m/z
KPVQSGPLK+XL_L
KVQAAQSEAK+XL_S
B_b_ion
B_y_ion
KVQAAQSEAK+XL_L
